# Pacific decadal oscillation and ENSO forcings of northerly low-level jets in South America

**DOI:** 10.1038/s41612-024-00852-6

**Published:** 2024-12-04

**Authors:** Ye Mu, Charles Jones, Leila M. V. Carvalho, Lulin Xue, Changhai Liu, Qinghua Ding

**Affiliations:** 1 Department of Geography, University of California Santa Barbara, Santa Barbara, CA USA; 2 Earth Research Institute, University of California Santa Barbara, Santa Barbara, CA USA; 3 NSF National Center for Atmospheric Research, Boulder, CO USA

**Keywords:** Atmospheric dynamics, Climate change

## Abstract

The hydrological cycle in South America during austral summer, including extreme precipitation and floods, is significantly influenced by northerly low-level jets (LLJs) along the eastern Andes. These synoptic weather events have been associated with three different types of LLJs (Central, Northern, and Andes) and are sensitive to remote large-scale forcings. This study investigates how tropical forcings related to El Niño/Southern Oscillation (ENSO) and Pacific Decadal Oscillation (PDO) regulate the duration and frequency of each LLJ type and their impacts on extreme precipitation. Our analysis reveals that ENSO and PDO are important in driving the variability of LLJs over the past 65 years. Specifically, the Central LLJ type is more prevalent during El Niño and Warm/Neutral PDO phases, leading to heightened extreme precipitation in southern South America. Conversely, La Niña years during Cold PDO phases tend to favor the Northern and Andes LLJs, which are associated with increased precipitation extremes in the western Amazon and southeastern South America. Central and Andes LLJs tend to persist longer during these favored conditions, causing more pronounced precipitation events in the areas under their influence. This study enhances our understanding of the influence of large-scale atmospheric forcings on the regional precipitation dynamics in South America.

## Introduction

Low-level jets (LLJs) are highly efficient in transporting heat, moisture, and momentum due to their strong intensity and the availability of abundant lower-atmosphere moisture. Originating from local-to-regional boundary layer processes, LLJs typically peak at nighttime and are observed across diverse climates^1,2^. They usually feature a horizontal extension ranging over hundreds to thousands of kilometers, influenced by topographic features and atmospheric pressure gradients. Vertically, they exhibit a pronounced wind speed maximum near the 850 hPa level, which facilitates enhanced moisture transport, particularly during nocturnal stable conditions^3–5^, In addition, LLJs are not only influenced by synoptic-to-large-scale atmospheric circulation patterns in their local area but also by remote forcing mechanisms^6–9^, Despite recent advancements, we still lack a comprehensive understanding of their local and remote forcing mechanisms and related impacts on precipitation beyond the synoptic time scale. Here, for brevity, we refer to nocturnal northerly low-level jets along the eastern Andes, which is the most essential feature of LLJs over South America, as simply LLJs.

In South America, three types of LLJs along the eastern Andes have been identified^10^. The Central type, also known as the South American low-level Jet (SALLJ), is located along the eastern slopes of the central Andes (Bolivia, Paraguay, northern Argentina), playing a vital role in precipitation and often triggering heavy rainfall and mesoscale convective systems (MCSs) in southeastern South America^4,11–16^, The Northern type, also known as Orinoco LLJ, is found in the northern Andes (northeastern Peru, Colombia, Venezuela) and brings Atlantic moisture inland, enhancing rainfall in the northern Andes and western Amazon^9,10,13,17–21^, In addition, Jones et al.^10^ identified the Andes (simultaneous occurrence in northern and central Andes), and Peru (short-lived and less frequent) types.

The interaction between upper-level wave trains and the subtropical jet has significant implications for the activity of the LLJ. The upper-level Rossby wave trains, often initiated by large-scale atmospheric phenomena such as the El Niño/Southern Oscillation (ENSO), propagate along the subtropical jet, modifying LLJ strength and position. During El Niño phases, these wave trains often strengthen the subtropical jet by amplifying zonal wind speeds and creating positive 200 hPa geopotential height anomalies, which enhance the meridional pressure gradient, driving stronger Central LLJ events^22^. Remote forcings from the equatorial Pacific on intraseasonal-to-interannual time scales influence the variability and evolution of LLJs^10^. On interannual time scales, for instance, ENSO plays an important role in linking LLJs and precipitation in South America. El Niño years increase the frequency and duration of Central LLJ events, which, in turn, extend the duration of precipitation events in South American subtropical region (SESA). In contrast, La Niña years favor an increase of Northern LLJ events and their duration, leading to greater persistence of precipitation in the central Andes and southwestern Amazon.

Other modes of low-frequency variability such as the Pacific Decadal Oscillation (PDO) and the Atlantic Multidecadal Oscillation (AMO) also have a significant impact on climate variability in South America (e.g.^7,23–27^). The PDO, for example, has been shown to influence the South American climate by modulating rainfall variability, which can lead to extreme climate conditions such as floods and multi-year droughts^8,24^, During the warm phase of the PDO, southeastern South America experiences positive precipitation anomalies, whereas negative precipitation anomalies occur in northern South America. In the cold PDO phase, the pattern reverses^28–30^. In addition, the warm and cold phases of the PDO can enhance the El Niño and La Niña phases and amplify their teleconnections^31^. During the warm phases of PDO and El Niño, there is an intensification of warm water conditions in the tropical Pacific, leading to further weakening of the trade winds and more extreme wet-dry conditions across various regions of the globe^32,33^, The AMO-negative phase can strengthen and displace the South Atlantic Convergence Zone, shifting its precipitation band further south. This displacement could enhance the Andes LLJs, particularly those directed toward southeastern Brazil, due to increased moisture transport and favorable large-scale atmospheric circulation patterns. However, during this phase, Central LLJ activity is often weakened, which may be attributed to changes in the meridional pressure gradient and altered moisture pathways that favor the Andes region^7,8^.

While previous studies have investigated how ENSO modifies the variability of LLJs in South America^4,5,9,10^, the relationships among ENSO, PDO, and LLJs have not been fully studied and understood. The main objective of this study is to decipher the role of large-scale modes on the variability of LLJs and precipitation extremes in South America on interannual-to-decadal time scales. Specifically, building upon Jones et al.^10^ this study aims to investigate the importance of the joint variability of ENSO and PDO in controlling the activity of the LLJ and precipitation in South America during austral summers. We use 65 years of reanalysis to characterize the LLJ variability and large-scale teleconnections associated with ENSO and PDO. Since the typical period of the AMO spans several decades, and only one complete cycle has been observed over the past 65 years, we do not examine its impacts. A particular emphasis is placed on the linkages among ENSO-PDO, LLJ activity in the northern and central Andes, and extreme precipitation across South America. Decadal changes in precipitation in South America are also assessed with a combination of reanalysis and observed gridded precipitation.

## Results

### Trends in LLJ intensity and frequency

**Fig. 1:**
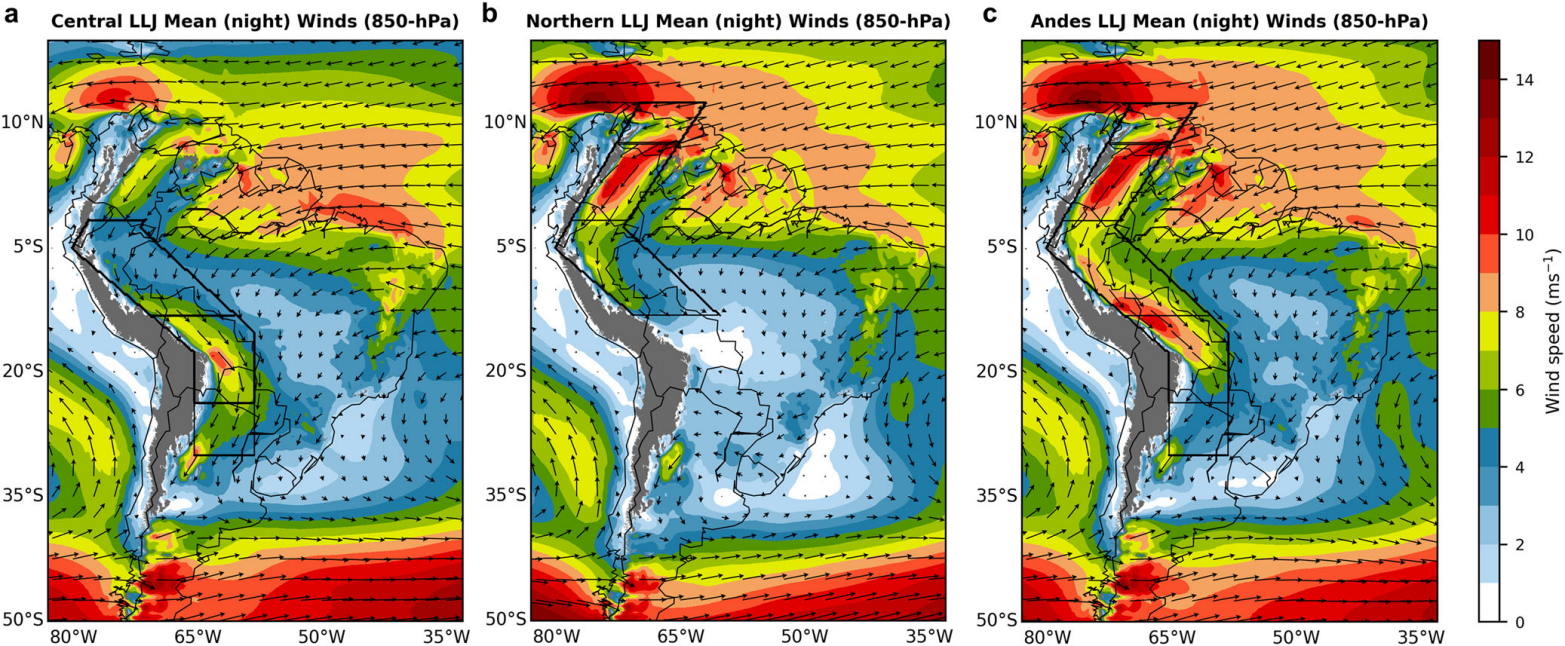
Northerly Low-level Jets. Composites of Mean DJF Nighttime Winds (850-hPa) and spatial domains for **a** Central, **b** Northern, and **c** Andes LLJ types in South America. The color shading indicates wind speed in meters per second with arrows depicting the direction and relative magnitude of the winds. Nighttime corresponds to 23-09 UTC (19:00–05:00 Bolivia local time). Black boxes show the spatial domain masks to identify LLJs.

The following analysis focuses on LLJs occurring in the northern and central parts of the Andes Mountains (see “Methods”) during austral summers (DJF). Fig. 1 shows the wind climatology at 850-hPa and the spatial extents of the Central, Northern, and Andes types. Note that in any given LLJ type day, the LLJ may not extend through all the spatial domain regions simultaneously (Fig. 1). During austral summers (1957–2022), the Northern and Central types account for 20% and 34.7% of the total LLJ cases, respectively. Moreover, consistent with Jones et al.^10^ the Andes type has the highest frequency (44.4%). The Peru LLJ type (0.9% frequency) identified in ref. ^10^ is excluded from this study since its frequency is much less than the other types. The three major LLJ types (Northern, Central, and Andes) account for 99% of all 4946 LLJ cases.

**Fig. 2:**
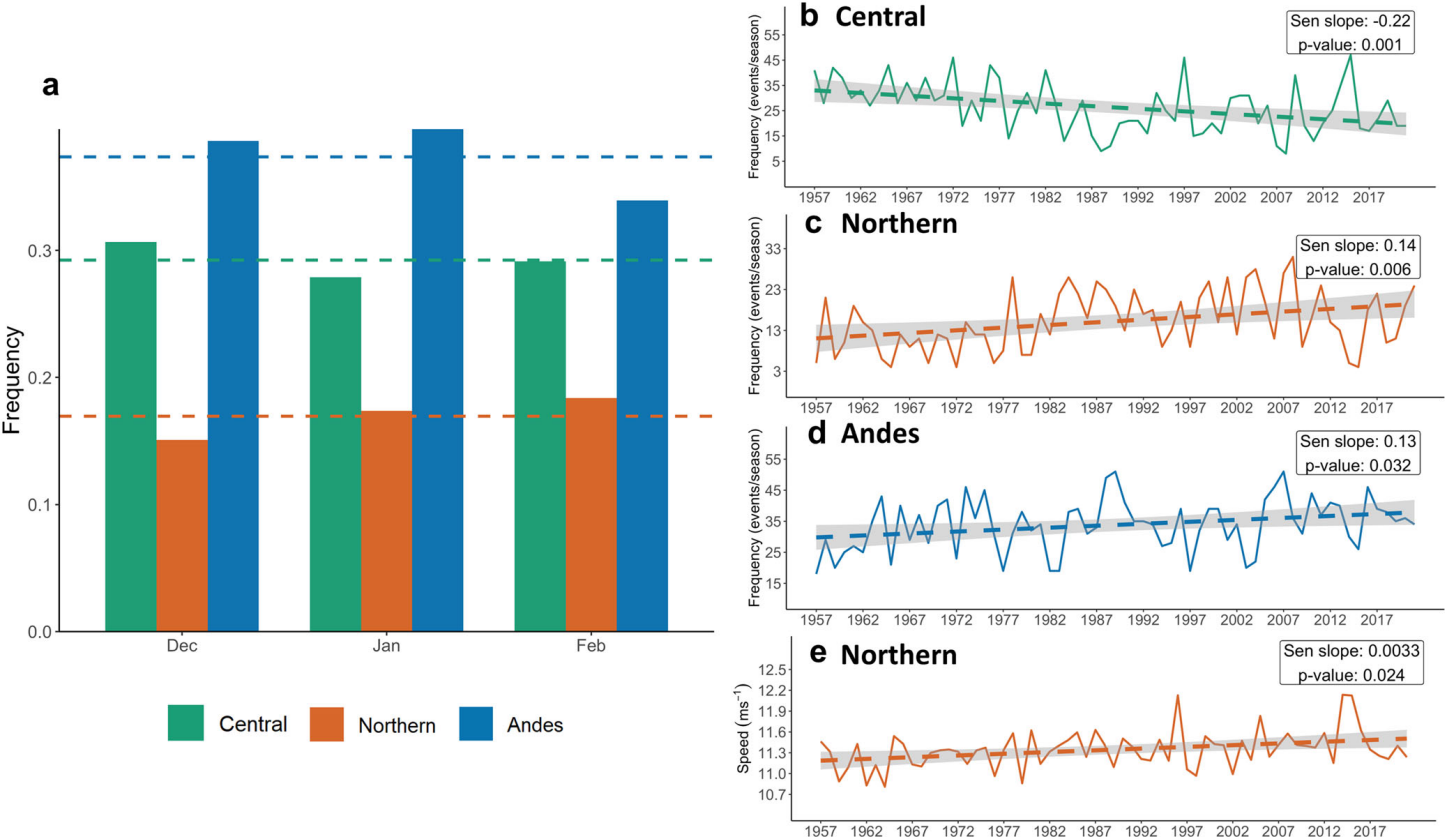
Frequency and wind speed trends of austral summer LLJs. **a** Bars represent monthly mean frequencies of three LLJ types. Dashed lines indicate the mean frequency of the LLJ types across all months. Time series of LLJ frequency for Central (**b**), Northern (**c**), and Andes (**d**) types from 1957–2022. **e** Time series of LLJ wind speeds for the Northern type from 1957 to 2022. Dashed lines show trends with gray shading representing confidence intervals. The statistical significance of the trends, determined using Mann–Kendall tests, is shown in the insets. Monthly LLJ frequency is calculated by dividing the number of LLJ days by the total number of days in each month.

The variability of the LLJ types during austral summer is shown in Fig. 2a and indicates that the Andes type is most frequent from December to February, with the Northern type being the least for all 3 months. The trends shown in Fig. 2 reflect changes in the frequency and intensity of LLJs over the 65-year period of the study. The observed trends provide a contextual background to understand the modulation of LLJ activity by ENSO and PDO phases. While we show trends in LLJ types (Fig. 2), we did not analyze trends in ENSO or PDO phases themselves, so any potential shifts in their frequency were not examined in this study. By identifying these trends, we can better interpret how ENSO and PDO influence LLJ behavior within the broader context of changing climatic conditions. Positive trends in frequency are statistically significant for the Northern and Andes types, but Central LLJs display a significant negative trend (Fig. 2b–d). Interestingly, the Northern LLJ type also shows a positive significant trend in wind speeds (850-hPa) (Fig. 2e). These results suggest that the frequency and intensity of LLJs in the northern Andes have both increased in the last 65 years, which is consistent with Jones^13^.

### The role of large-scale remote forcings

To examine the role of large-scale forcings connected with trends in frequency and intensity of the LLJ, we employ the National Oceanic and Atmospheric Administration (NOAA) ENSO and PDO seasonal indices along with ERA5 reanalysis. As the warm and cold phases of the PDO can enhance El Niño and La Niña episodes, respectively, and thus amplify their effects^33,34^, we examined the in-phase and out-of-phase ENSO-PDO combinations. The relatively long data records and large number of LLJ cases facilitate an investigation of interannual-to-decadal large-scale forcings on the LLJs and give rise to more robust conclusions.

**Fig. 3:**
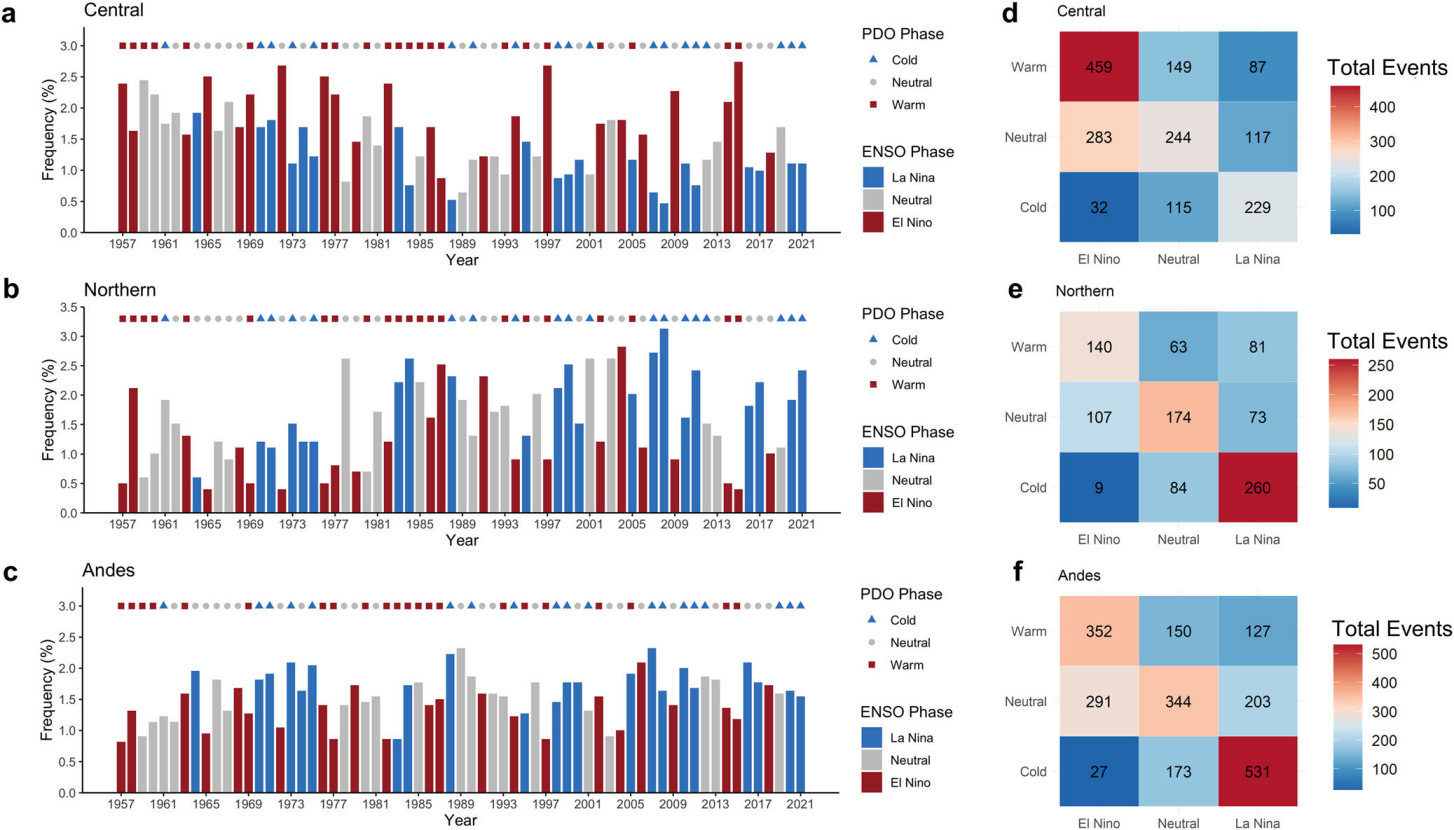
LLJ frequencies during ENSO and PDO phases. Annual frequencies (%) (DJF) of **a** Central, **b** Northern, and **c** Andes LLJ types during warm, neutral, and cold ENSO-PDO phases, and total events (days) for **d** Central, **e** Northern, and **f** Andes across all ENSO-PDO combinations. Frequency ratios in (**a**–**c**) are represented in red bars for El Niño, blue bars for La Niña, and gray bars for neutral phases. LLJ frequency ratios are calculated by dividing the number of LLJ days in each DJF season by the total LLJ days from 1957 to 2022. Red squares correspond to warm PDO, gray circles to neutral PDO, and blue triangles to cold PDO phases.

The variability of LLJs on interannual-to-decadal time scales is shown in Fig. 3, where the temporal distributions of the three LLJ types are color-coded according to warm (red), neutral (gray), and cold (blue) phases of ENSO and PDO to illustrate the joint impacts of ENSO and PDO. Note that there is only 1 year of El Niño+Cold cases, and this combination is not discussed further. For the Central LLJ, the highest frequency is observed during El Niño+Warm PDO, where 459 events were recorded, accounting for 48.3% of the total LLJ events for that ENSO-PDO joint phase (Fig. 3d). In contrast, the lowest frequency for the Central LLJ occurs during La Niña+Cold with 229 events The Northern LLJ displays a different pattern, with the highest frequency during La Niña+Cold, recording 260 events (Fig. 3e). The Andes LLJ, on the other hand, shows its highest frequency during La Niña+Cold phases, with 531 events, representing 52.1% of the total for that joint phase (Fig. 3f). The lowest frequency of the Andes LLJ is during El Niño+Warm PDO, which is 37% of the total for that joint phase.

Overall, the analysis reveals distinct patterns in the frequencies of Central, Northern, and Andes LLJs during different ENSO-PDO phases. The Central LLJ shows a strong dominance during El Niño conditions, particularly when coupled with a warm PDO, while the Northern LLJ exhibits higher frequency during La Niña and cold conditions, with reduced occurrences during El Niño. The Andes LLJ exhibits a clear preference for La Niña phases, especially when combined with cold PDO, highlighting the variability in the behavior of these LLJs in response to large-scale climatic oscillations.

**Fig. 4:**
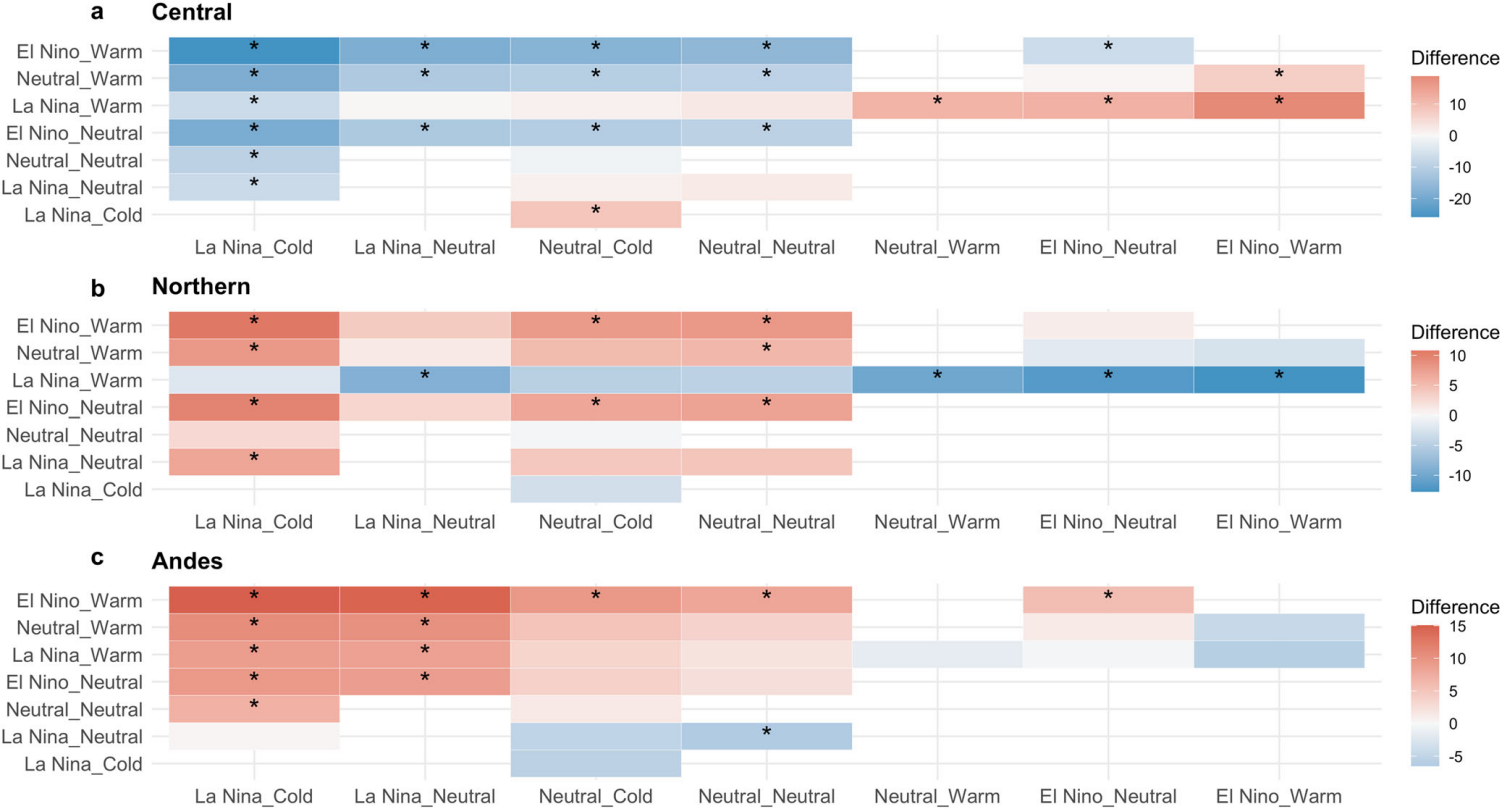
Heatmaps of differences in LLJ occurrences during ENSO-PDO phase combinations. Heatmaps display the differences in percentages of events in **a** Central, **b** Northern, and **c** Andes LLJs for various combinations of ENSO and PDO phases. The *x* axis and *y* axis represent different phase combinations, with colors indicating the magnitude and direction of the differences in occurrences: blue signifies that the occurrence is lower for the phase combination on the *x* axis compared to the phase combination on the *y* axis, while red signifies higher occurrences for the phase combination on the *x* axis compared to the phase combination on the *y* axis. Asterisks (*) denote statistically significant differences determined using two-tailed Z-tests (*P* < 0.05).

Figure 4 displays heatmaps of differences in proportions of LLJ occurrences (Central, Northern, and Andes) for various joint ENSO-PDO phases. For the Central LLJ, there are significantly higher occurrences of events during the El Niño+Warm/El Niño +Neutral compared to La Niña+Cold, La Niña +Warm, La Niña +Neutral, Neutral+Cold, and Neutral+Neutral, as indicated by the red color and asterisks (Fig. 4a). However, El Niño+Warm has a statistically higher frequency than both El Niño+Neutral and Neutral+Warm. Additionally, El Niño+Neutral is not higher than Neutral+Warm. The Central type also has significantly lower occurrences during the La Niña+Cold compared to all other phase combinations. This suggests that the La Niña +Cold is less favorable for the occurrence of Central LLJ compared to other phases, particularly those involving El Niño or neutral ENSO with varying PDO phases. The Northern LLJ shows a contrasting pattern with a predominance of red cells, showing statistically significant higher occurrences for La Niña+Cold/Warm compared to El Niño+Neutral/Warm, La Niña+Neutral, and Neutral+Warm (Fig. 4b). For the Andes type, La Niña+Cold exhibits higher occurrences compared to El Niño+Neutral/Warm, La Niña +Warm, and Neutral+Warm/Neutral, as indicated by the red cells and asterisks. In addition, La Niña+Neutral also shows significant differences but lacks Neutral+Neutral, but both La Niña+Cold and Neutral PDO are statistically more frequent than La Niña+Warm.

**Fig. 5:**
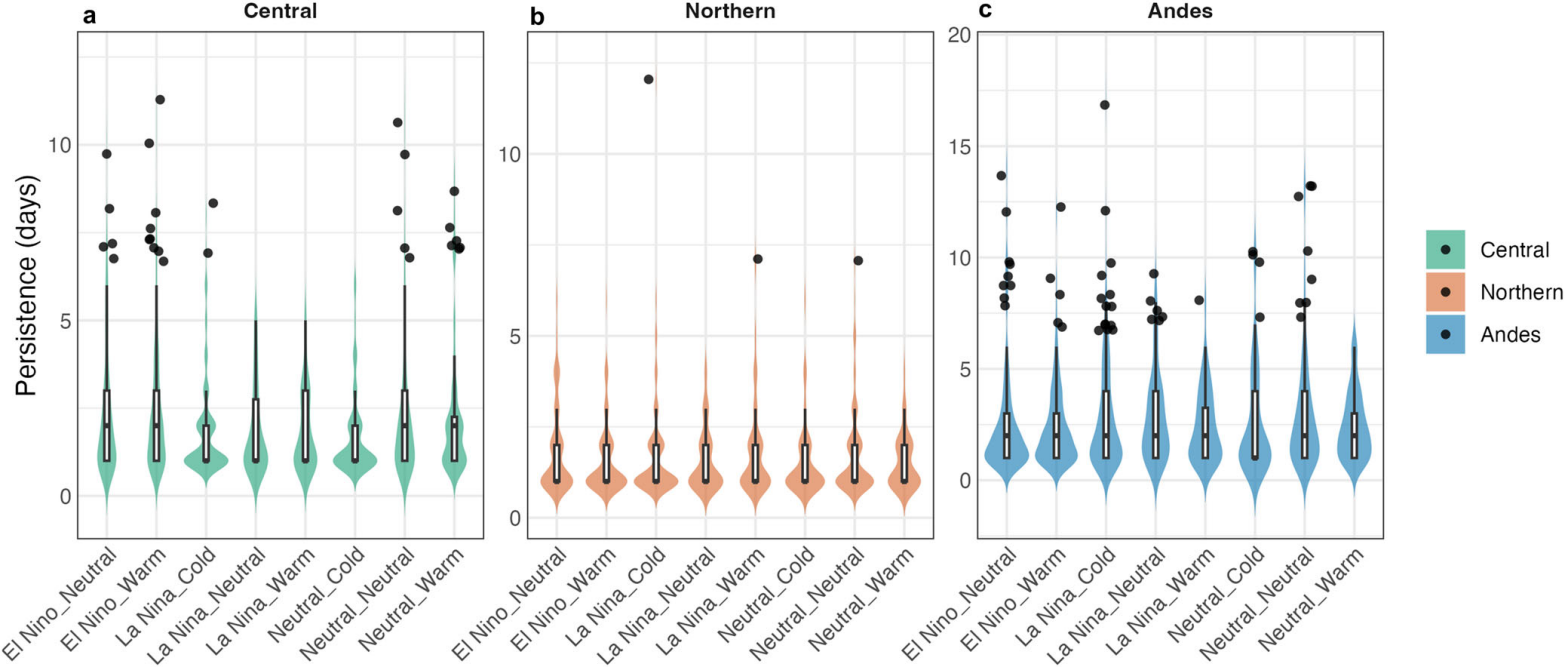
Persistence of LLJs across different ENSO and PDO phases. The violin plots show the distribution of persistence (in days) for each ENSO-PDO combination, with **a** Central, **b** Northern, and **c** Andes types represented by different colors. The boxplots inside the violins show the median and interquartile range, while the black dots represent outliers. The violin plot includes areas slightly below zero due to kernel density estimation smoothing, although no actual data values fall below zero.

Figure 5 illustrates the distribution of persistence for different LLJ types (Central, Northern, Andes) during various combinations of ENSO and PDO phases. The violin plots summarize the data distribution, with embedded boxplots highlighting the median and interquartile range, and outliers marked as black dots. For the Central type, the majority of the values cluster around lower persistence days, with occasional outliers indicating longer persistence of events (Fig. 5a). Notably, the El Niño+Warm exhibits more persistent LLJs, as indicated by the higher medians and the presence of several outliers reaching above 10 days. However, Neutral+Neutral and Neutral+Warm also exhibit long Central LLJ persistence. The Northern type shows relatively low variability across the different ENSO and PDO phases (Fig. 5b). The persistence values are generally lower, with fewer outliers compared to the Central and Andes types. This indicates that none of the phase combinations result in significantly higher persistence for Northern LLJs. For the Andes type, while several phase combinations, such as La Niña+Neutral and Neutral+Neutral, show comparable median persistence, the La Niña + Cold phase stands out with the highest number of outliers (Fig. 5c). Our findings suggest that the changes in frequency and persistence are more pronounced under the different ENSO and PDO phases. However, the mean wind speed, used as a proxy for LLJ intensity, did not show a clear pattern of variation in relation to these phases, and those results are not included here.

**Fig. 6:**
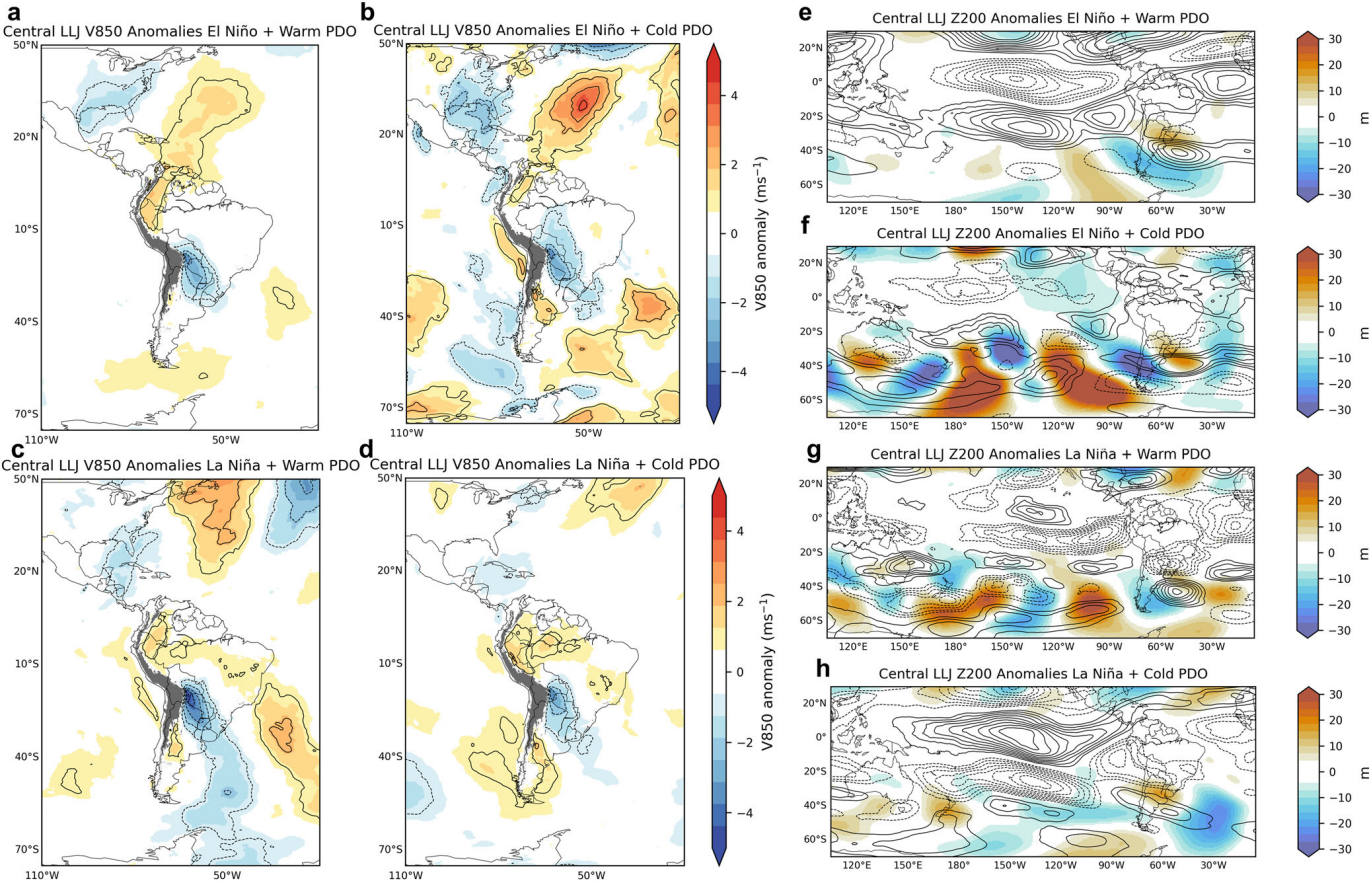
Composites of austral summer low-level (850-hPa) meridional winds and upper-level anomalies during Central LLJ and ENSO-PDO phases. The left column shows 850-hPa meridional wind (V850) anomalies (m s^−1^) during Central LLJ and **a** El Niño plus Warm PDO, **b** El Niño plus Cold PDO, **c** La Niña plus Warm PDO and **d** La Niña plus Cold PDO phases. The right column shows 200-hPa geopotential height (m) (Z200) and 200-hPa zonal wind (U200) anomalies during the Central LLJ type and **e** El Niño plus Warm PDO, **f** El Niño plus Cold PDO, **g** La Niña plus Warm PDO and **h** La Niña plus Cold PDO phases. Z200 anomalies are shown in colors. Positive (negative) U200 anomalies are shown in solid (dashed) contours (1 m s^−1^ interval), with zero contour omitted. Anomalies are statistically significant at a 95% confidence level.

**Fig. 7:**
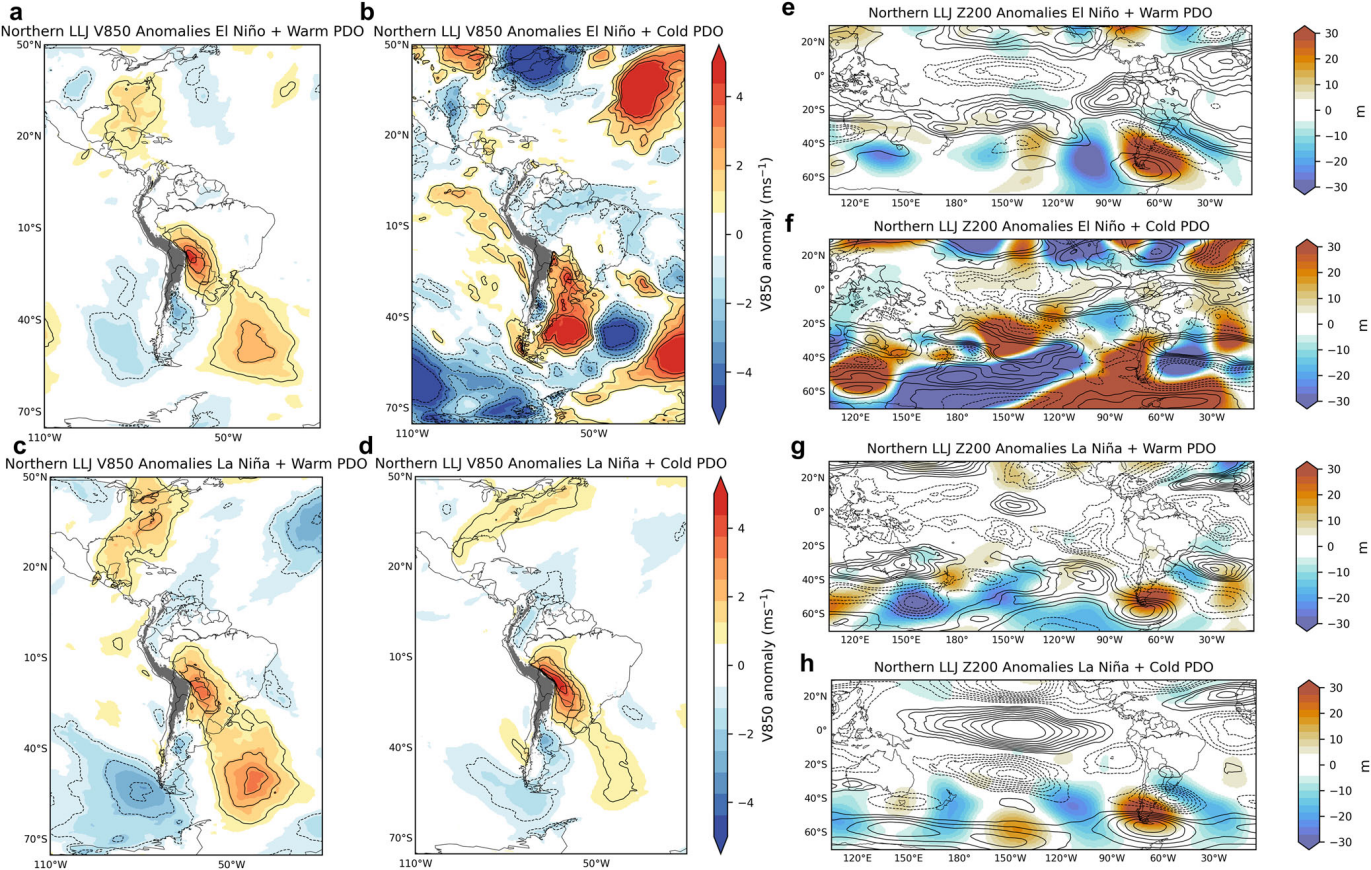
Composites of austral summer low-level (850-hPa) meridional winds and upper-level anomalies during Northern LLJ and ENSO-PDO phases. The left column shows 850-hPa meridional wind (V850) anomalies (m s^−1^) during Northern LLJ and **a** El Niño plus Warm PDO, **b** El Niño plus Cold PDO, **c** La Niña plus Warm PDO and **d** La Niña plus Cold PDO phases. The right column shows 200-hPa geopotential height (m) (Z200) and 200-hPa zonal wind (U200) anomalies during the Northern LLJ type and **e** El Niño plus Warm PDO, **f** El Niño plus Cold PDO, **g** La Niña plus Warm PDO and **h** La Niña plus Cold PDO phases. Z200 anomalies are shown in colors. Positive (negative) U200 anomalies are shown in solid (dashed) contours (1 m s^−1^ interval), with zero contour omitted. Anomalies are statistically significant at a 95% confidence level.

**Fig. 8:**
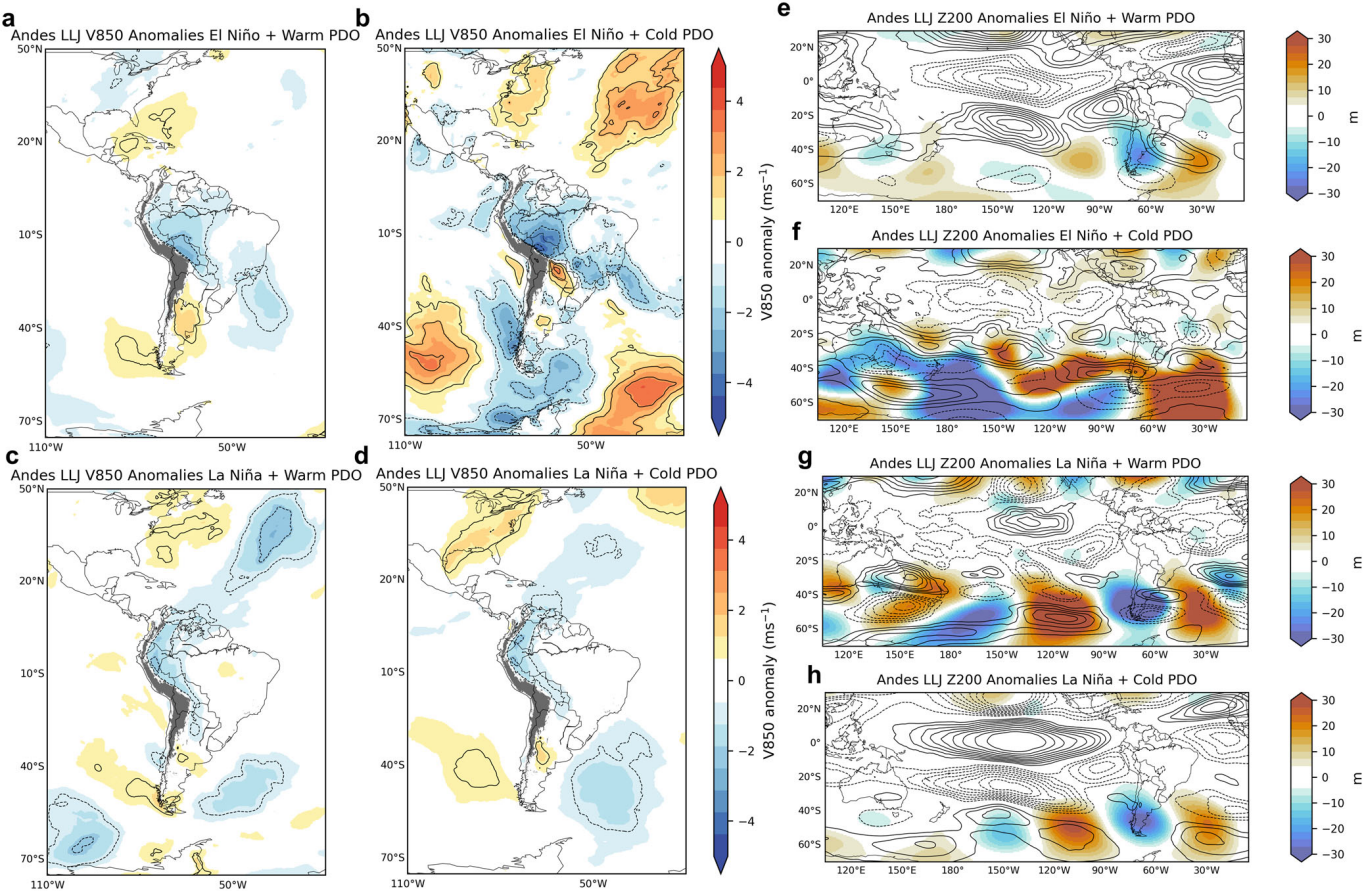
Composites of austral summer low-level (850-hPa) meridional winds and upper-level anomalies during Andes LLJ and ENSO-PDO phases. The left column shows 850-hPa meridional wind (V850) anomalies (m s^−1^) during Andes LLJ and **a** El Niño plus Warm PDO, **b** El Niño plus Cold PDO, **c** La Niña plus Warm PDO and **d** La Niña plus Cold PDO phases. The right column shows 200-hPa geopotential height (m) (Z200) and 200-hPa zonal wind (U200) anomalies during the Andes LLJ type and **e** El Niño plus Warm PDO, **f** El Niño plus Cold PDO, **g** La Niña plus Warm PDO and **h** La Niña plus Cold PDO phases. Z200 anomalies are shown in colors. Positive (negative) U200 anomalies are shown in solid (dashed) contours (1 m s^−1^ interval), with zero contour omitted. Anomalies are statistically significant at a 95% confidence level.

To further examine large-scale atmospheric circulation patterns, Figs. 6–8 show composites of meridional winds (850-hPa, V850), zonal winds (200-hPa, U200), and geopotential height (200-hPa, Z200) anomalies during the favored phases of ENSO and PDO combinations for each LLJ type. For the Central LLJ, during El Niño+PDO phases (Figs. 6a, b and S2a), V850 anomalies generally display southerly components over northern South America and northerly components over southern South America, which may reduce the formation of Northern LLJs. The upper-level Z200 anomalies show a wave train pattern extending from the Pacific into South America, potentially modulating the subtropical jet stream. Positive U200 wind anomalies are observed crossing the central Andes and interacting with positive/negative Z200 anomalies in subtropical South America during El Niño+Warm/Neutral PDO, possibly enhancing the subtropical jet. During La Niña, the upper-level conditions typically feature stronger zonal flows with positive anomalies over the central Pacific, which may stabilize the atmosphere and reduce convective activity, potentially reducing the Central LLJ occurrences (Fig. 6g, h). However, during La Niña+Warm PDO (Fig. 6c), V850 anomalies show strong northerly winds over central South America, indicating that individual LLJ events may be more intense during this phase. Despite these wind anomalies, the overall frequency of Central LLJ events tends to be higher during El Niño + Warm PDO phases.

For the Northern LLJ, V850 wind anomalies show extended southerly components over central South America during Warm PDO phases (Fig. 7a, c). This configuration indicates stronger southward wind flow, which might influence the typical northward transport of moist air associated with the South American Monsoon System (SAMS). La Niña combined with Cold PDO phases (Fig. 7c, d) tends to show northerly wind anomalies over northern South America, which could strengthen the southward flow. The upper-level anomalies are marked by negative anomalies over the central Andes which may weaken the subtropical jet and suppress the occurrence of the Central type (Fig. 7g, h). During La Niña, the Andes type has negative V850 anomalies in the North Atlantic and flows along the eastern slopes of the northern and central Andes (Fig. 8c, d). In the upper levels (Fig. 8e–h), the zonal propagation of Z200 anomalies seems to have less influence on the subtropical jet, though La Niña+Warm PDO favors northerly winds, potentially supporting Andes LLJ formation (Fig. 8c). The La Niña+Cold PDO combination also reinforces northerly wind patterns, particularly in the northern Andes domain.

**Fig. 9:**
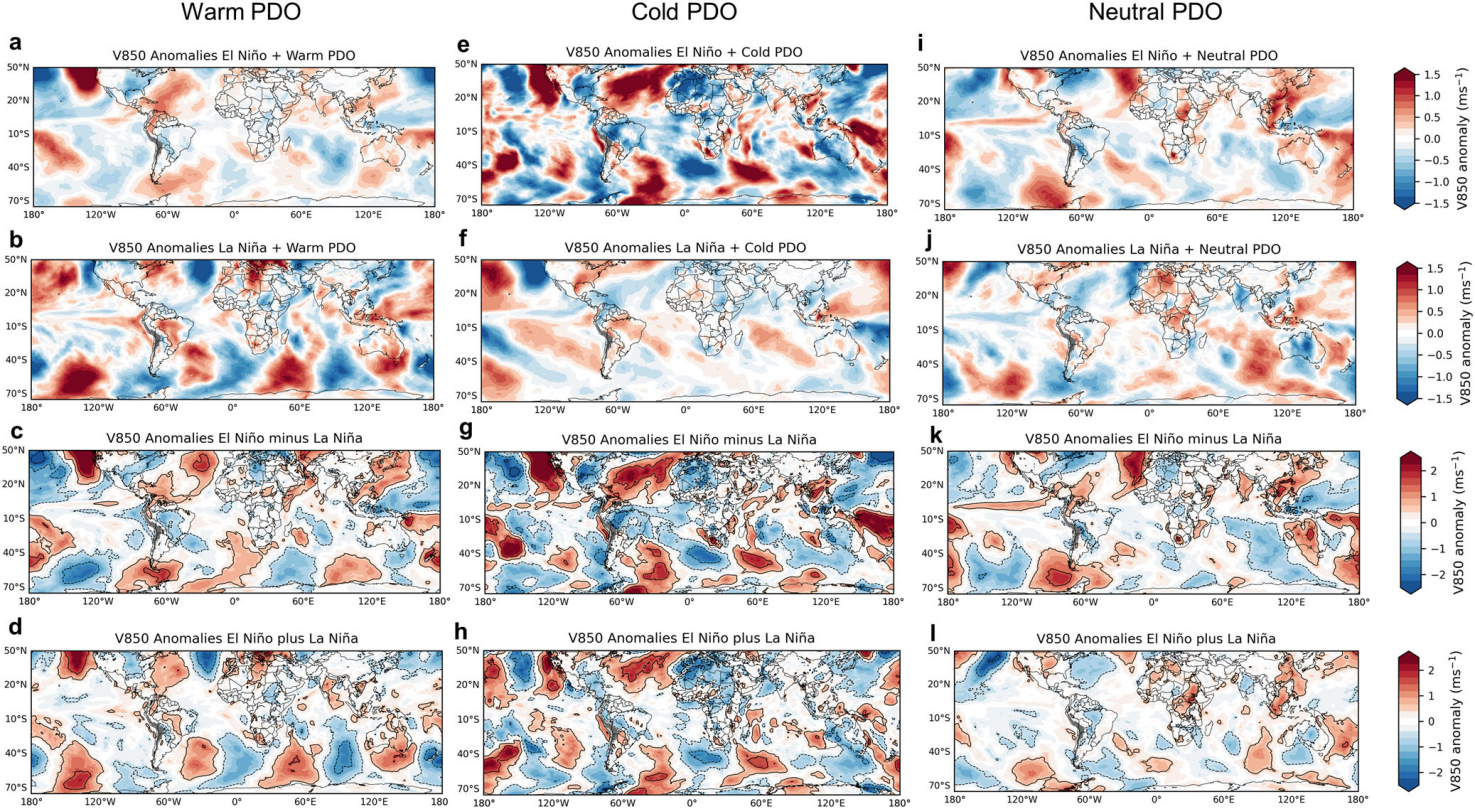
Composites of 850-hPa meridional wind (V850) anomalies (ms^−1^). The left column shows composites of V850 anomalies during Warm PDO and **a** El Niño and **b** La Niña phases. The middle column shows composites of V850 anomalies during Cold PDO and **e** El Niño and **f** La Niña phases. The right column shows composites of V850 anomalies during Cold PDO and **i** El Niño and **j** La Niña phases. Panels (**c**, **g**, **k**) and (**d**, **h**, **l**) show La Niña minus El Niño composites (linear component), and La Niña plus El Niño composites (nonlinear component), respectively. Solid (dashed) contours in the linear (**c**, **g**) and nonlinear (**d**, **h**) panels indicate positive (negative) anomalies statistically significant at a 95% confidence level. The contour interval is 1 ms^−1^.

It has been demonstrated that ENSO modifies the mean atmospheric state and, thus, rainfall and temperature variability in South America (e.g.^35–38^). Additional understanding is gained by compositing low-level meridional winds under different combinations of ENSO-PDO phases to examine their in-phase and out-of-phase contributions to changes in the low-level meridional circulation (Fig. 9). Note that composites are calculated by averaging V850 anomalies during all days in the respective PDO and ENSO phases regardless of the occurrence of LLJs.

During the El Niño+Warm PDO phase, the V850 anomalies enhance northerly winds over the central Andes, promoting the formation of the Central LLJ type (Fig. 9a). When La Niña coincides with Warm PDO, the V850 anomalies show enhanced northerly winds over the northern Andes (Fig. 9b). The differences in composites (Fig. 9c, d) suggest that the in-phase component, i.e., El Niño minus La Niña (Fig. 9c), dominates the out-of-phase component, i.e., El Niño plus La Niña Warm PDO phases (Fig. 9d). The El Niño+Cold PDO phase is less favorable for any specific LLJ type, creating a complex and variable atmospheric environment (Fig. 9e). The Cold PDO tends to moderate the typical El Niño effects, leading to less pronounced atmospheric stability and moisture transport^39^. In contrast, changes in the mean atmospheric state during La Niña years occurring in Cold PDO phases favor the occurrence of the Northern and Andes types (Fig. 9f). The differences in composites (Fig. 9g, h) also indicate that the linear component dominates. Furthermore, ENSO teleconnections in low-level circulation appear to be stronger over South America during the Cold PDO. During La Niña plus Cold PDO phases, the differences in the anticyclonic activity in the North Atlantic Ocean (Fig. 9b, f) and the waveguides in the Southern Hemisphere are enhanced (Fig. 9c, g), creating more favorable conditions for the Northern and Andes LLJs.

When the PDO is neutral during El Niño conditions, enhanced northerly winds over the central Andes continue to support the Central LLJ (Fig. 9i). Similarly, during La Niña+Neutral PDO, the V850 anomalies continue to show northerly winds over the northern Andes, but it is weaker compared to the La Niña+Cold PDO phase. Comparing these phases, El Niño phases, especially when combined with a warm PDO, consistently favor the Central LLJ in the central Andes. La Niña phases, particularly with a cold PDO, enhance northerly winds, favoring the Northern and Andes LLJs. Neutral PDO phases moderate these effects, maintaining the influence of ENSO-driven patterns on LLJ formation.

### Precipitation extremes during LLJ types

Moisture transport by LLJs is critical in modulating precipitation variability in South America as well as the formation and maintenance of MCS (e.g.^11,12,15,16^). For the Northern LLJ, La Niña years favor longer precipitation events than El Niño years. In contrast, El Niño years favor persistence with longer duration than in La Niña years during Central types^10^. The previous results indicate that the Central type is more likely to occur during El Niño+Warm/Neutral PDO, while the occurrence of the Northern type is favored by La Niña+Cold/Warm PDO phases. Therefore, the specific purpose is to investigate how the combination of ENSO-PDO phases influences the frequency of extreme precipitation events in South America, particularly through their modulation of LLJ behavior. We hypothesize that these climate oscillations not only impact the occurrence of LLJ types but also directly influence the frequency of extreme precipitation.

**Fig. 10:**
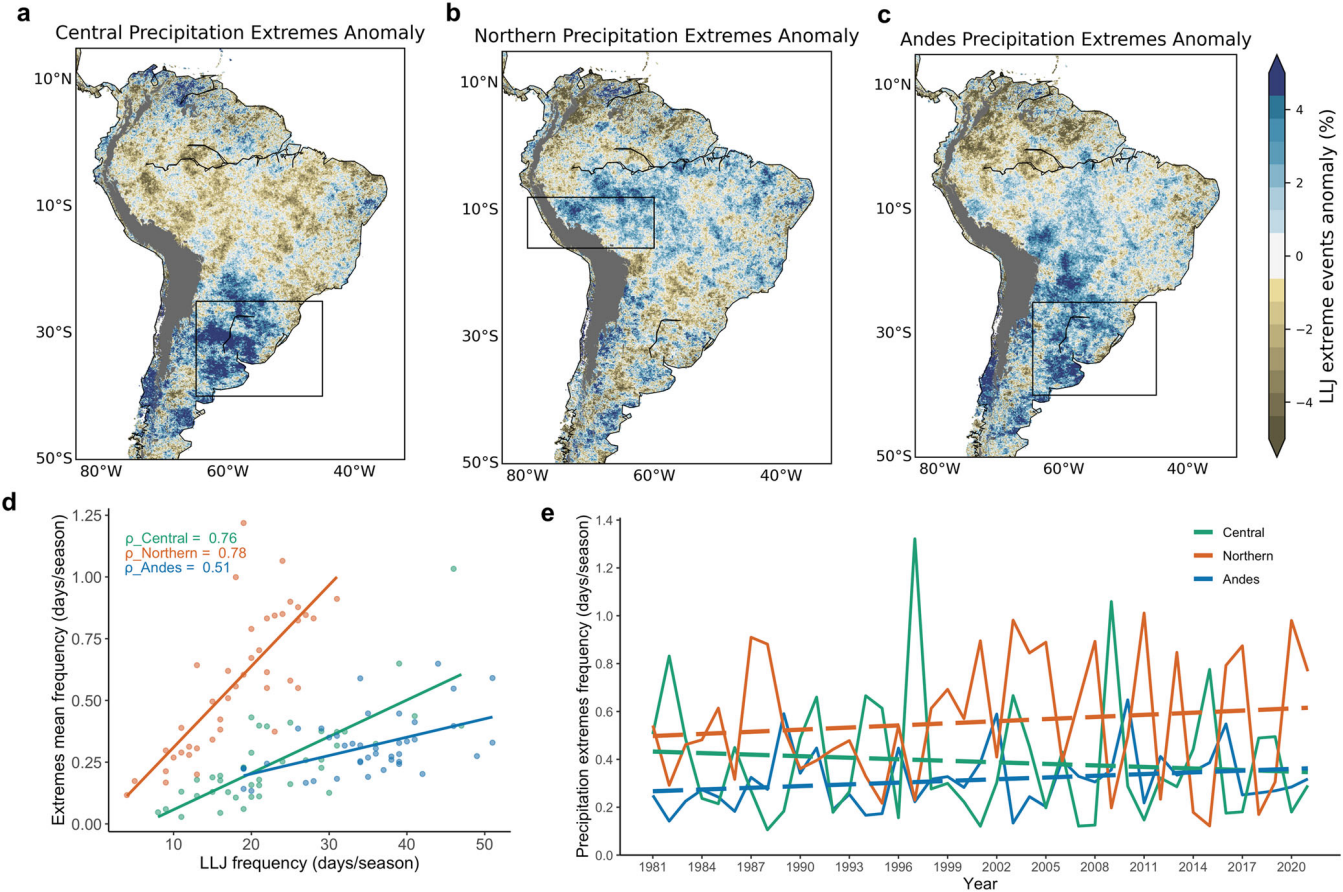
Precipitation extreme anomalies during LLJ types. Daily DJF precipitation extreme anomalies (%) during **a** Central, **b** Northern, and **c** Andes LLJs. Black boxes in (**a**–**c**) are located downstream from the respective exit regions of the three LLJ types. **d** Correlation (*ρ*) between the frequency of Central (green), Northern (orange), and Andes (blue) LLJ types and frequency of precipitation extremes (days/season) from 1981 to 2021. **e** Time series of frequency of precipitation extremes (days/season) for each LLJ type. The dashed line shows trends of precipitation extremes for each LLJ type.

The importance of LLJs in modulating precipitation variability is further investigated by calculating precipitation extreme anomalies in the SESA and western Amazon Basin (black boxes in Fig. 10). During Central LLJs, positive precipitation extreme anomalies are spatially coherent in the SESA region (Fig. 10a), consistent with the influence of the Central type on MCS formation^16^. In contrast, during Northern LLJs (Fig. 10b), a large area of precipitation extreme anomalies extends over the Amazon Basin and western Brazil. While there are small pockets of precipitation extremes in the SESA domain, there is a clear reduction of extremes. Precipitation extreme anomalies during Andes types (Fig. 10c) show positive anomalies over parts of the Amazon Basin, Central Brazil, and SESA. To examine the relationship between extreme precipitation and LLJs, Fig. 10d shows scatterplots of the frequency of precipitation extreme anomalies and LLJs frequency. The relationships are separated by Central, Northern, and Andes types and show positive correlations, especially for the Northern LLJ type and precipitation extremes in the northwestern Amazon. Lastly, Fig. 10e shows the seasonal frequency of precipitation extreme anomalies during 1981–2021 associated with each LLJ type. An increase in precipitation extremes over the western Amazon is related to the increase in the Northern LLJ activity (compare Fig. 2).

**Fig. 11:**
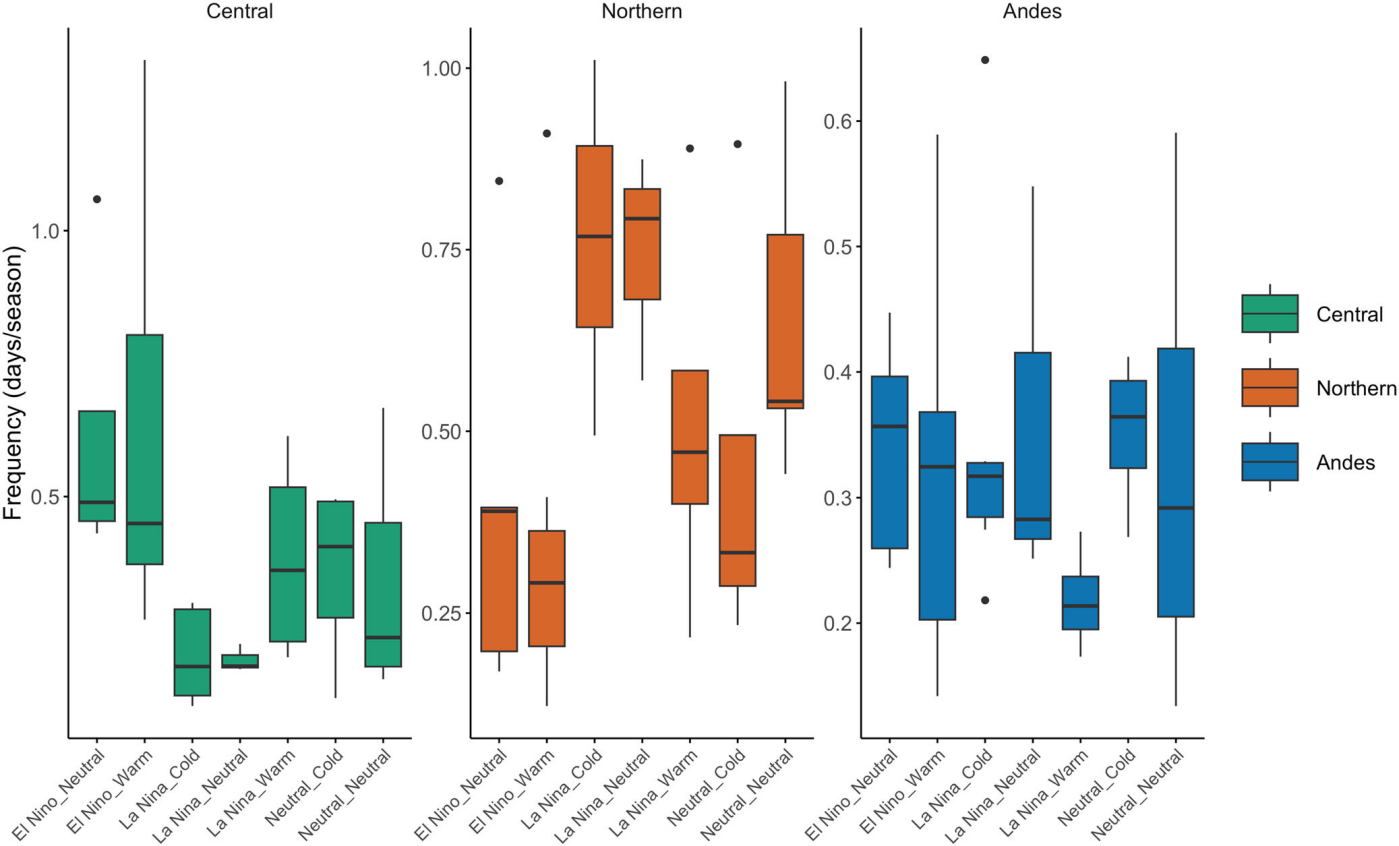
Seasonal frequency of extreme precipitation events of LLJs during ENSO-PDO phases. The boxplots represent the distribution of frequencies during different phases of the ENSO and PDO from 1981 to 2021. El Niño plus Cold PDO is excluded due to insufficient samples during this period. The green, orange, and blue colors correspond to **a** Central, **b** Northern, and **c** Andes LLJs, respectively. The central line within each box plot indicates the median frequency, while the edges of the box mark the interquartile range (IQR). Whiskers extend to the 1.5*IQR, and outliers are represented by individual points.

Figure 11 shows the seasonal frequency of extreme precipitation events across different types (Central, Northern, and Andes) under varying combinations of the ENSO-PDO. Due to the small sample size for the El Niño+Cold and Neutral+Warm during the CHIRPS periods, these combinations are not shown in the plot. For the Central Andes, the occurrence of extreme precipitation events is most frequent during El Niño phases, particularly when the PDO is neutral or warm (Fig. 11a). The Central LLJ positive influence on precipitation is evident from the higher median frequencies of extreme events during El Niño+Warm/Neutral phases. In the Northern Andes, extreme precipitation events are favored during La Niña phases, especially when combined with a Cold PDO (Fig. 11b). During La Niña plus Cold PDO phases, the meridional circulation is enhanced along the eastern Andes, strengthening the Northern LLJ and leading to increased precipitation extremes in the exit region. The Andes type shows a complex relationship with extreme precipitation (Fig. 11c). These events are not favored by any single ENSO-PDO phase combination strongly but exhibit variability depending on the interactions between the phases.

## Discussion

This study uses 65 years of ERA5 reanalysis to investigate the variability of LLJs along the eastern Andes on interannual-to-decadal scales. The research focuses on the joint variability of ENSO and PDO phases and their influence in controlling the activity of LLJs in the northern and central Andes. The results reveal distinct patterns in LLJ behavior contingent on ENSO and PDO phases, demonstrating the complex interactions between these large-scale forcings and LLJ dynamics. El Niño years during Warm and Neutral PDO phases were found to favor high frequency and persistence of the Central LLJ type while inhibiting the formation of LLJs in the northern Andes. Conversely, La Niña years during Cold PDO phases enhance the frequency and persistence of the Northern and Andes LLJ types and suppress the Central LLJ type. The influence of out-of-phase ENSO and PDO combinations on LLJs is notably weaker, highlighting the critical role of phase synchronization in modulating LLJ activity.

The frequency and intensity of the Northern LLJ type show positive trends in the sixty-five years of ERA5 reanalysis. In contrast, the Central type shows a statistically significant negative trend in the frequency of occurrence but not in its intensity. While the frequency of the Andes type also shows a positive increase, the increase in its intensity is not statistically significant. The increase in frequency and intensity of LLJs in the northern Andes is consistent with Jones, who used a different methodology of LLJs identification and found similar positive trends^13^. The temporal distributions of LLJ types differentiated by ENSO and PDO phases (Fig. 3) show a higher number of joint occurrences of La Niña and Cold PDO phases since the early 2000s than in the preceding period. These conditions favor the activity of the Northern type and suppress the occurrence of the Central LLJ. Jones found negative trends in wind shear anomalies over the central Andes, which, therefore, create less favorable conditions for the Central LLJ type^13^.

LLJs transport significant amounts of warm and moist air from the equatorial Atlantic and Amazon Basin and, consequently, supply the moisture for the formation of MCSs^14–16^. This study contextualizes the findings within broader precipitation extreme variability across South America. In summary, the Central LLJ is most active and promotes extreme precipitation during El Niño phases with a Warm or Neutral PDO. The Northern LLJ is favored during La Niña phases with a Cold PDO, leading to increased precipitation extremes in the northern Andes regions. The Andes LLJ, representing simultaneous occurrences of LLJs in both central and northern Andes, is influenced by La Niña phases, especially with a Cold PDO, contributing to extreme precipitation events across a broader area. These findings align with the understanding that ENSO and PDO phases modulate LLJ activity and extreme precipitation patterns in South America.

Zhang et al. reported a noticeable multidecadal positive moistening trend during the twentieth century over southeastern South America, followed by a decadal negative trend in rainfall starting around 2000^40^. They concluded that the decrease in decadal rainfall is primarily attributed to the observed strengthening of the Pacific trade winds, which is likely associated with natural Pacific decadal variability^40^. While earlier studies^41,42^ identified positive precipitation trends in southern Brazil of SESA, our analysis using ERA5 and CPC data shows no significant positive precipitation trends in SESA from 1957 to 2022 (Supplementary Fig. 4). In addition, the CHIRPS dataset reveals no significant trends in extreme events during Central and Andes LLJ types over the past 40 years. Both Central and Andes LLJs influence precipitation in the SESA region, but their contributions and trends differ. The Central LLJ, with a significant decreasing trend in frequency, is less frequent during recent decades, corresponding to fewer El Niño events during Warm/Neutral PDO phases. Conversely, the Andes LLJ shows a significant increasing trend in frequency, primarily driven by La Niña phases with Cold PDO, contributing more to recent extreme precipitation events. However, Andes LLJs exhibit low correlations with SESA precipitation extremes (0.51, Fig. 10d), indicating that while they play roles in modulating precipitation, other factors also contribute to precipitation variability in the region.

Changes in the activity of LLJs in the northern and central Andes can be viewed as resulting from changes in large-scale atmospheric circulation on interannual-to-decadal time scales. During El Niño events, the warming of the central and eastern Pacific leads to a weakening of the Walker circulation, characterized by weaker trade winds and an eastward shift of the convection zone^30,33,43,44^. This alteration in atmospheric circulation results in anomalous westerly winds at lower levels and the strengthening of the subtropical jet, disrupting the typical easterly flow and enhancing the formation of the Central LLJ^23,45,46^. In contrast, during La Niña events, cooler SSTs in the central and eastern Pacific strengthen the Walker circulation, enhancing the easterly trade winds^40,47–49^. These enhanced easterlies can extend into the northern and eastern Andes, promoting the formation of the Northern LLJ. In addition, the Cold PDO phase, characterized by cooler sea surface temperatures (SST) in the North Pacific, can reinforce La Niña conditions by further enhancing the trade winds and altering the subtropical jet^23,50^. This explanation is consistent with the changes in the mean atmospheric state during La Niña years in Cold PDO phases (Fig. 9f), which favor an increase in the activity of the Northern and Andes LLJs.

Some studies indicated that the cooling trends observed in the tropical Pacific might be largely attributed to internal climate variability mechanisms, notably the Pacific Decadal Variability (PDV), as opposed to a direct result of anthropogenic factors. Moreover, it is anticipated that this cooling will eventually stabilize at a neutral state before transitioning into a period of warming^40,51^. If this transitioning scenario materializes to more frequent El Niño years occurring during Warm/Neutral PDO phases, our analysis indicates a potential intensification of the Central LLJ type, while simultaneously reducing the activity of the Northern and Andes LLJs. These changes are likely to exacerbate strengthened precipitation variability in South America, leading to more frequent extreme rainfall events in SESA and inducing drier conditions within the southwestern Amazon Basin. To fully comprehend these complex interactions, there is a pressing need to investigate deeper into the connections between large-scale atmospheric forcings, the behavior of LLJs, and precipitation variability in South America. Assessing the fidelity of global climate models in representing these processes is a critical next step. It is essential to improve climate models to more accurately simulate the observed relationships and, thus, enhance predictive capabilities and mitigation strategies.

## Methods

The European Centre for Medium-Range Weather Forecasts reanalysis v5 (ERA5)^52^ (available from 1940 to 2023) was used to identify LLJs, climatology, variability, and large-scale forcings. ERA5 was used with 0.25° × 0.25° latitude/longitude resolution during austral summers (December 1st–Feb 28, 1957–2022) (DJF). Variables used include u-velocity, v-velocity at 850-hPa and 700-hPa pressure levels, geopotential height at 200-hPa, and precipitation. Precipitation was used with daily resolution and all other variables at the hourly resolution. We found a discontinuity in ERA5 data in representing the frequency and intensity of all LLJ types from 1940 to 1956 and, therefore, the analysis excluded data prior to November 1957 (see examples in Supplementary Fig. 5). LLJ definition was based on: (a) nighttime (23-09 UTC) wind speed (850-hPa) greater than the 75th percentile of the monthly frequency distribution; (b) wind shear between 850-hPa and 700-hPa greater than the 75th percentile; and (c) spatial extent is identified by contiguous regions of wind speeds greater than 10 m/s. The detailed LLJ identification method is described in ref. ^10^. The Northern LLJ occurs along the eastern Andes from Venezuela to Peru, while the Central LLJ occurs in Bolivia and Paraguay. The Andes LLJ occurs simultaneously in the northern and central Andes. The Peru LLJ type occurs primarily in central eastern Peru and is characterized by its short-lived nature and small spatial extent. The Peru-type LLJ, being the least frequent, was not included in this study. LLJ frequency refers to the total days of LLJ occurrence for each year from December to February, and the LLJ intensity refers to the mean wind speed (850-hPa) in the LLJ spatial extent where the wind speed is greater than 10 m/s. Figure 1 shows the annual summertime time series of frequency and intensity as well as linear trends for each type. Trends are based on Mann–Kendall tests statistically significant at 95% confidence.

ENSO classification was based on the Climate Prediction Center, National Centers for Environmental Prediction (https://origin.cpc.ncep.noaa.gov/) from 1957 to 2022. The Pacific Decadal Oscillation (PDO) index data was obtained from the National Centers for Environmental Information (NCEI) (https://www.ncei.noaa.gov/access/monitoring/pdo/) and covered the period from December 1957 to February 2022. The mean (μ) and standard deviation (σ) of the PDO index for the December to February period were calculated. A classification function was developed to categorize the PDO index values into three phases: Warm, Cold, and Neutral. The criteria were based on the mean and standard deviation of the PDO index, using a 0.5 standard deviation threshold: Warm Phase: PDO >μ + 0.5σ; Cold Phase: PDO <μ − 0.5σ; and Neutral Phase: μ − 0.5σ ≤ PDO ≤ μ + 0.5σ. The dominant phase for each season year was determined by identifying the phase with the highest count within that season. To discriminate the joint effects of PDO and ENSO phases on the types of LLJ, they are combined for eight joint phases, including EN+ Warm PDO, EN+Cold PDO, EN+Neutral PDO, LN+Warm PDO, LN+ Cold PDO, LN+Neutral PDO, Neutral ENSO+ Warm PDO, Neutral ENSO+ Cold PDO. For each pair of phase combinations, we constructed a contingency table and performed a Two-Tailed Z-test to compare the LLJ occurrences. The proportions of occurrences were calculated for each combination, and the difference in these proportions between the two combinations was computed. A *P* value threshold of 0.05 was used to determine significance, with *P* values below this threshold indicating significant differences.

To focus on the extratropic Rossby wave trains affecting South America^35^, daily ERA5 200-hPa geopotential height was filtered to retain variations between 2.5 and 30 days and eastward propagating 1–15 zonal wavenumbers (Figs. 6–8). To show circulation anomalies during LLJs, the annual cycle was removed from the time series of U and V at 850-hPa and 200-hPa by subtracting the climatology determined with the first two harmonics. Basic states comparison (Fig. 9) uses the V850 anomalies during El Niño and La Niña combined with Warm, Cold, and Neutral PDO phases during DJF. Anomalies statistical significance is based on two-sample *t* test at 95% confidence level.

Daily precipitation data over South America were obtained from the ERA5 precipitation (0.25°, 1957–2023), CPC Global Unified Gauge-Based Analysis of Daily Precipitation (0.5°, 1979–2023)^53^, and the Climate Hazards Group InfraRed Precipitation with Station data (CHIRPS)^54^. Supplementary Fig. 4 presents the aggregated precipitation data for the South American subtropical region (SESA). It is derived by first summing the daily precipitation amounts over the DJF season and then calculating the average of these sums across the SESA region. While all three precipitation datasets have inherent biases, ERA5 and CPC show trends opposite to those reported in the literature over the SESA region (Supplementary Fig. 4), we based our results on CHIRPS data at 0.05° resolution from 1981 to 2022. Extreme precipitation events were defined as the 95th percentile of daily precipitation. To show the LLJ influence on precipitation extremes, we defined the frequency of LLJ precipitation extremes anomaly as:

$$\begin{array}{l}{{\boldsymbol{Anomaly}}}_{{\boldsymbol{LLJ}}\;{\boldsymbol{precipitation}}\;{\boldsymbol{extremes}}}=\left(\frac{{\#}{\boldsymbol{LLJ}}\; {\boldsymbol{extremes}}\; {\boldsymbol{events}}}{{\#}{\boldsymbol{LLJ}}\; {\boldsymbol{precipitation}}\; {\boldsymbol{events}}}\right){\times}{\bf{100}}{\boldsymbol{ \% }}\\\qquad\qquad\qquad\qquad\qquad\qquad\qquad\qquad\qquad-\left(\frac{{\#}{\boldsymbol{No}}{{\_}}{\boldsymbol{LLJ}}\; {\boldsymbol{extremes}}\; {\boldsymbol{events}}}{{\#}{\boldsymbol{No}}{{\_}}{\boldsymbol{LLJ}}\; {\boldsymbol{precipitation}}\; {\boldsymbol{events}}}\right){\boldsymbol{\times }}{\bf{100}}{\boldsymbol{ \% }}\end{array}$$

In this equation, ‘#‘ denotes the number of events, and LLJ refers to the type of jet (Central, Northern, and Andes). Given that the number of LLJ events is only a subset of all precipitation events, we computed the anomaly as the difference between the ratio of LLJ extreme events and the ratio of non-LLJ extreme events. The resulting precipitation extremes anomaly is then multiplied by 100 and expressed as a percentage.

## Supplementary information

SUPPLEMENTAL MATERIAL

## Data Availability

ERA5 data are available at the Copernicus Climate Change Service (C3S) Climate Date Store (10.24381/cds.bd0915c6). CPC data are available from (https://psl.noaa.gov/data/gridded/data.cpc.globalprecip.html). CHIRPS data are available from (https://data.chc.ucsb.edu/products/CHIRPS-2.0/).

## Code availability

Interactive Data Language (IDL) computer code was used for LLJ identification and geopotential height filtering. All figures in this paper were produced on the NCAR Cheyenne server in Python and the source codes can be obtained upon request to the corresponding author.
