# Supplementary material for: Pacific decadal oscillation and ENSO forcings of northerly low-level jets in South America
Source: NPJ Clim Atmos Sci. 2024 Dec 4;7(1):297. doi: 10.1038/s41612-024-00852-6 (PMC11618067; doi:10.1038/s41612-024-00852-6)
Supplement: Supplementary file 1 — SUPPLEMENTAL MATERIAL [file 41612_2024_852_MOESM1_ESM.pdf]

Supporting Information for

Pacific Decadal Oscillation and ENSO Forcings of Northerly Low-level Jets in South America

Ye Mu[*1], Charles Jones[13], Leila Carvalho[13], Lulin Xue[2], Changhai Liu[2], Qinghua Ding[13]

[1] Department of Geography, University of California Santa Barbara, CA 93106, USA.

[2] NSF National Center for Atmospheric Research, Boulder, CO, USA

[3] Earth Research Institute, University of California Santa Barbara, CA 93106, USA.

Contents of this file

Supplementary Figure 1

Supplementary Figure 2

Supplementary Figure 3

Supplementary Figure 4

Supplementary Figure 5

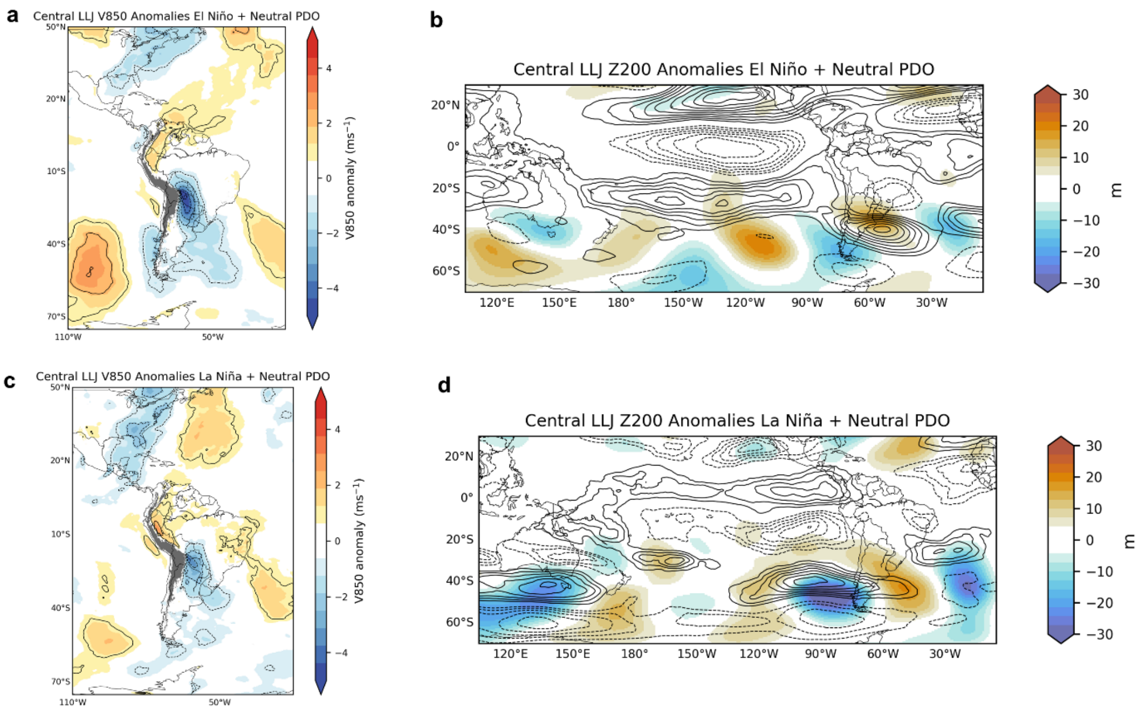

Supplementary Figure 1. Composites of austral summer meridional winds (850-hPa) and upper-level anomalies during Central LLJ and ENSO-Neutral PDO phases. The left column shows 850-hPa meridional wind (V850) anomalies (m s^{-1}) during Central LLJ and (a) El Niño plus Neutral PDO and (c) La Niña plus Neutral PDO phases. The right column shows 200-hPa geopotential height (m) (Z200) and 200-hPa zonal wind (U200) anomalies during the respective LLJ type and ENSO plus Neutral PDO phases. Z200 anomalies are shown in colors. Positive (negative) U200 anomalies are shown in solid (dashed) contours (1 m s-1 interval), with zero contour omitted. Anomalies are statistically significant at a 95% confidence level.

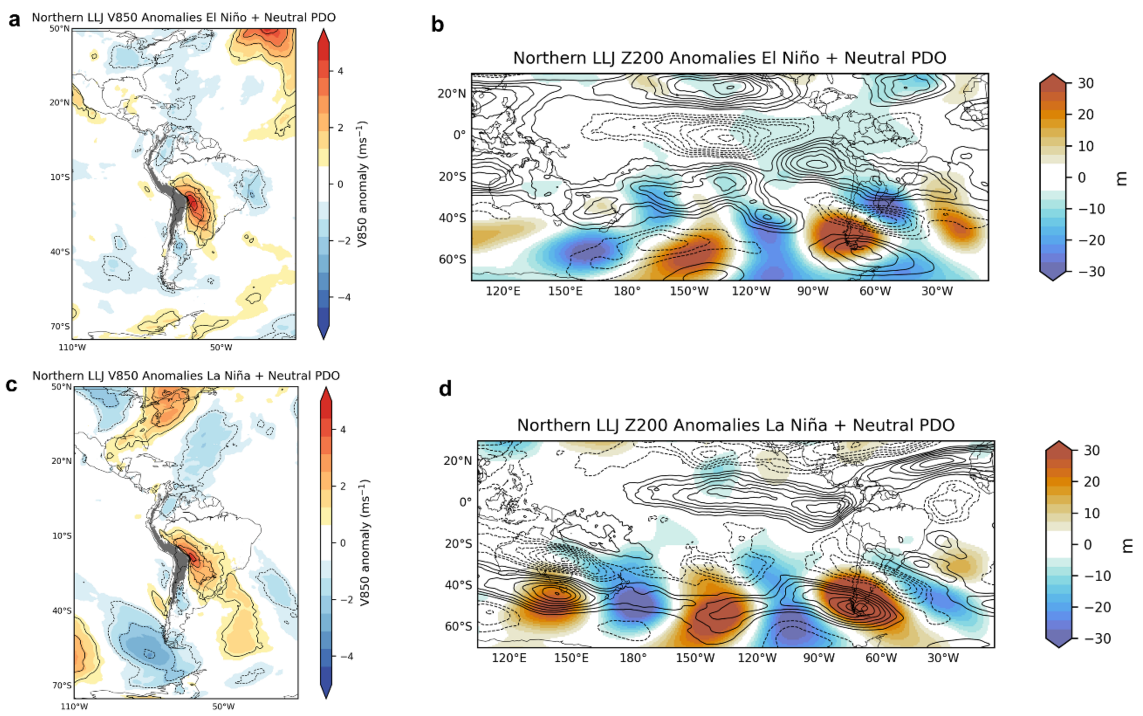

Supplementary Figure 2. Composites of austral summer meridional winds (850-hPa) and upper-level anomalies during Northern LLJ and ENSO-Neutral PDO phases. The left column shows 850-hPa meridional wind (V850) anomalies (m s^{-1}) during Northern LLJ and (a) El Niño plus Neutral PDO and (c) La Niña plus Neutral PDO phases. The right column shows 200-hPa geopotential height (m) (Z200) and 200-hPa zonal wind (U200) anomalies during the respective LLJ type and ENSO plus Neutral PDO phases. Z200 anomalies are shown in colors. Positive (negative) U200 anomalies are shown in solid (dashed) contours (1 m s-1 interval), with zero contour omitted. Anomalies are statistically significant at a 95% confidence level.

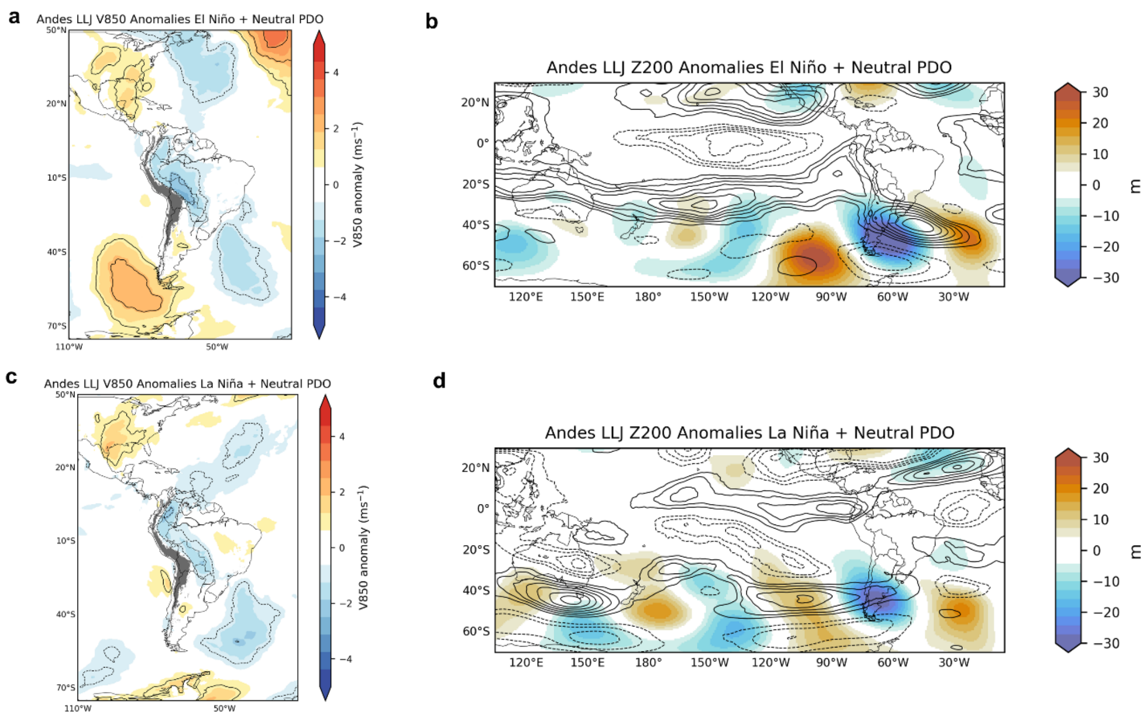

Supplementary Figure 3. Composites of austral summer meridional winds (850-hPa) and upper-level anomalies during Andes LLJ and ENSO-Neutral PDO phases. The left column shows 850-hPa meridional wind (V850) anomalies (m s^{-1}) during Andes LLJ and (a) El Niño plus Neutral PDO and (c) La Niña plus Neutral PDO phases. The right column shows 200-hPa geopotential height (m) (Z200) and 200-hPa zonal wind (U200) anomalies during the respective LLJ type and ENSO plus Neutral PDO phases. Z200 anomalies are shown in colors. Positive (negative) U200 anomalies are shown in solid (dashed) contours (1 m s-1 interval), with zero contour omitted. Anomalies are statistically significant at a 95% confidence level.

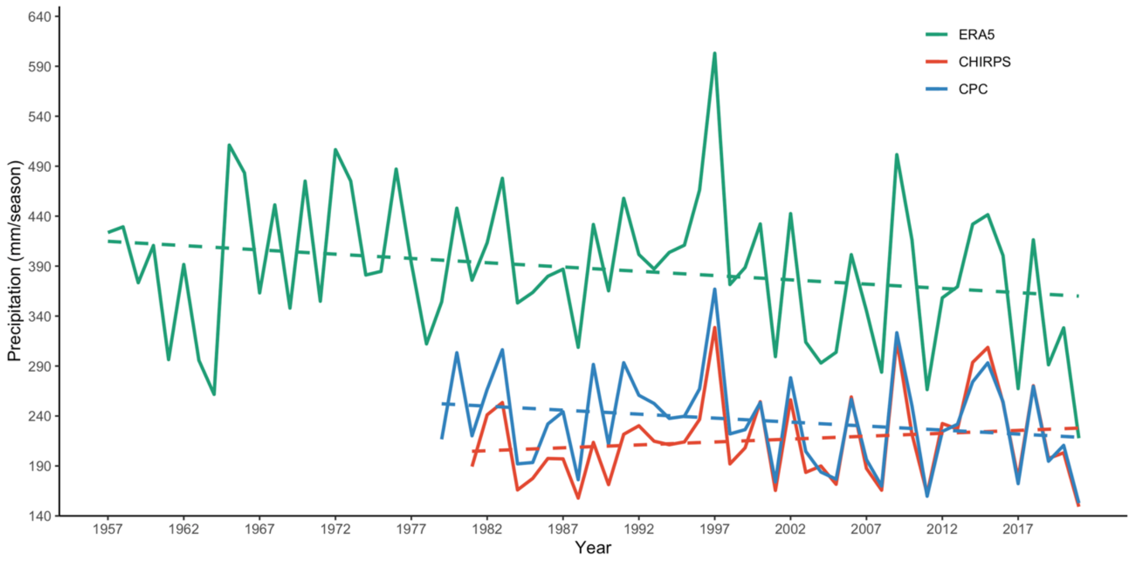

Supplementary Figure 4. SESA wet season (DJF) precipitation from 1957 to 2021 from ERA5, CHIRPS and CPC daily data.

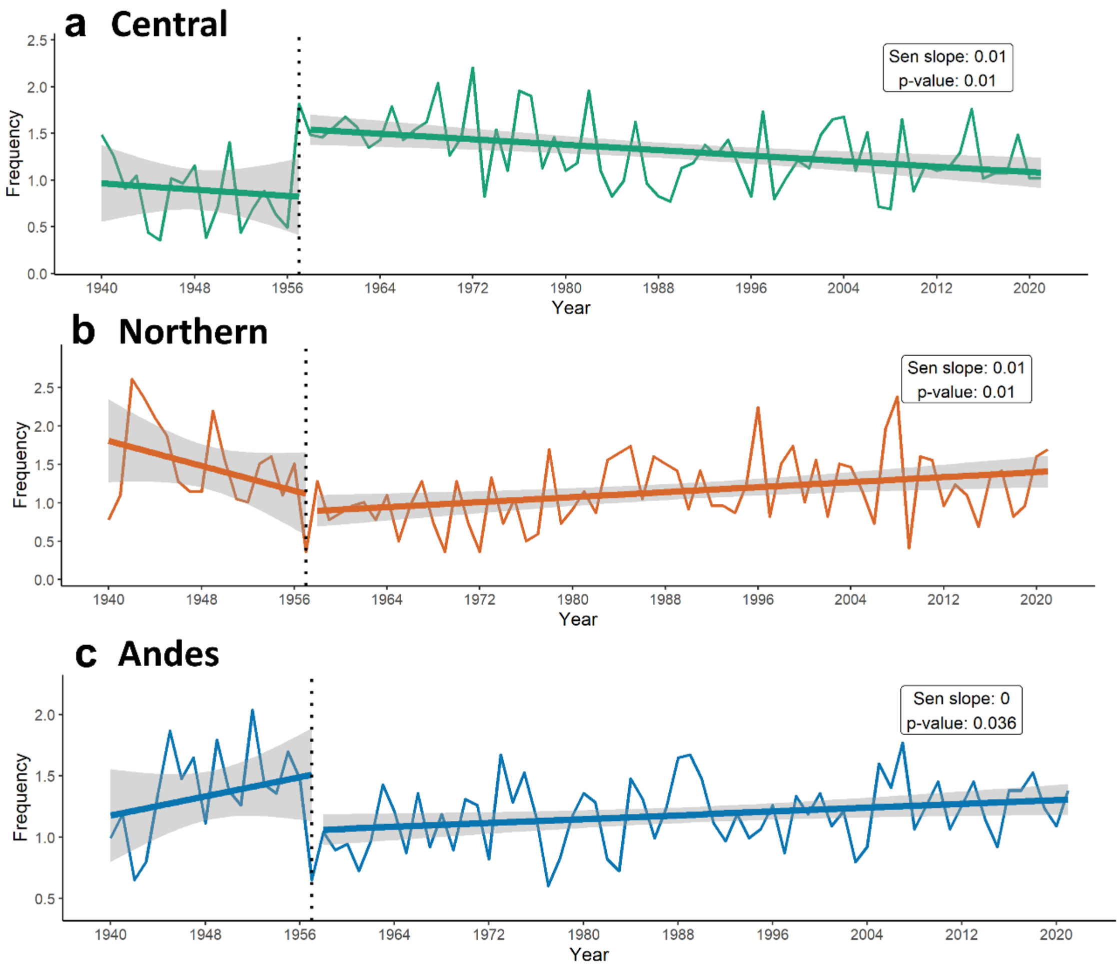

Supplementary Figure 5. Discontinuity in the frequency of Austral Summer for LLJs. Time series of LLJ frequency for Central (b), Northern (c), and Andes (d) types from 1940-2022. The vertical dashed line at 1957 highlights the discontinuity. Solid lines show trend lines, with grey shading representing the confidence intervals. Statistical significance of the trends, determined using Mann-Kendall tests, is shown in the insets. Monthly LLJ frequency is calculated by dividing the number of LLJ days by the total number of days in each month.
